# Updates on Anti-Obesity Medications in Children and Adolescents

**DOI:** 10.3390/children12101390

**Published:** 2025-10-15

**Authors:** Mostafa Salama, Doha Hassan, Seema Kumar

**Affiliations:** 1 Department of Pediatric and Adolescent Medicine, Mayo Clinic, 200 1st St SW, Rochester, MN 55905, USA; salama.mostafa@mayo.edu (M.S.); hassan.doha@mayo.edu (D.H.); 2 Division of Pediatric Endocrinology, Department of Pediatric and Adolescent Medicine, Mayo Clinic, 200 1st St SW, Rochester, MN 55905, USA

**Keywords:** children, obesity, treatment, anti-obesity medications

## Abstract

Childhood obesity has assumed epidemic proportions; it results from a complex interplay of genetic, physiological, socioeconomic, and environmental factors. It is associated with numerous short- and long-term health complications, including cardiometabolic and psychosocial consequences. While lifestyle modifications remain the cornerstone of treatment, their overall impact is modest. As a result, pharmacotherapy is increasingly recommended as an adjunct in selected pediatric patients, particularly those with severe or refractory obesity. In recent years, several anti-obesity medications, including glucagon-like peptide-1 (GLP-1) receptor agonists, have shown promise in pediatric populations, particularly adolescents. However, the pharmacologic options remain limited for younger children. This review summarizes current evidence on the efficacy and safety of available anti-obesity medications in children and adolescents, offering practical, age-based strategies to guide the appropriate clinical use of these medications as part of a comprehensive obesity-management plan.

## 1. Introduction

Obesity is a chronic disease arising from the complex interplay of genetic, biological, socioeconomic, and environmental influences [1]. Genetics account for 40–75% of the variability in obesity [1]. Social determinants such as education, income, and community environment, along with adverse childhood experiences, can disrupt the metabolic and epigenetic processes that regulate body weight [2]. Environmental factors—such as unhealthy dietary patterns, limited physical activity, and inadequate sleep—further promote weight gain. The list of additional contributors includes prenatal and early-life exposures, certain medications, endocrine disorders, gut microbiome alterations, and hypothalamic injuries, all of which may impair energy homeostasis and metabolic regulation [3].

Over the past several decades, the prevalence of pediatric obesity has increased markedly; it now affects approximately one in five children in the United States [4,5]. This rising trend extends beyond older adolescents to include younger age groups, with a particularly alarming increase observed in the rates of severe and extreme obesity, which are associated with a subsequent increase in cardiovascular risk [3,4,5,6].

The escalating burden of pediatric obesity has led to significant advancements in the pharmacotherapeutic options for pediatric obesity. Lifestyle modifications remain the cornerstone of obesity treatment [7]. The evidence supports intensive health behavior and lifestyle treatment (IHBLT) as the most effective behavioral intervention for pediatric obesity. IHBLT typically involves ≥ 26 contact hours delivered over 3 to 12 months by a multidisciplinary team, focusing on dietary modification, physical-activity promotion, and behavioral strategies to support sustainable lifestyle changes [8]. However, adherence to such programs is often limited, with high attrition rates which are attributed to factors such as unrealistic expectations regarding the magnitude and timing of weight loss, the substantial time commitment required, and financial or logistical barriers to sustained participation [9,10].

Given the modest efficacy of lifestyle interventions alone [11], pharmacotherapy is increasingly recognized as an important adjunct in the treatment of pediatric obesity, particularly in cases of severe or refractory obesity [12]. Most anti-obesity medications, including glucagon-like peptide-1 (GLP-1) receptor agonists and extended-release phentermine–topiramate (Qsymia), are currently approved by the United States Food and Drug Administration (FDA) for those 12 years and older [13,14,15]. However, treatment options for younger children remain limited, even though early intervention could reduce future cardiometabolic consequences.

This review examines current pharmacological options for pediatric obesity by age group, focusing on approved therapies for older children and adolescents, and potential options for younger children with severe obesity.

## 2. Pharmacological Treatment of Pediatric Obesity in Children Aged < 12 Years

Pharmacologic options for treating obesity in children under 12 are limited; currently setmelanotide is the only FDA-approved medication in this age group, specifically approved for Bardet–Biedl syndrome or monogenic obesity due to specific genetic mutations [16,17]. Lifestyle modifications remain the primary approach, with medications being regarded as a supplementary option, especially when related comorbidities are present [7]. Such decisions should be made collaboratively with families, following a thorough discussion of the potential benefits, risks, and side-effect profiles of these medications.

### 2.1. Topiramate

Topiramate is approved for the treatment of epilepsy in children aged 2 years and older, and for migraine prophylaxis in those 12 years and above [18,19,20]. While not approved for weight management in children under 12, it is sometimes used in the context of an off-label use for obesity, due to its appetite-suppressing effects [21].

**Figure 1:**
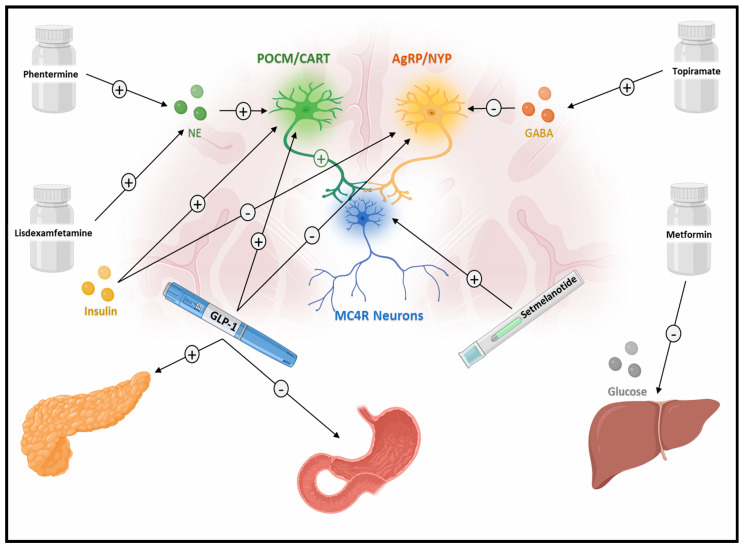
Appetite-suppressing effects of anti-obesity pharmacotherapies in children and adolescents. The diagram illustrates the proposed mechanisms by which commonly used pharmacologic agents contribute to weight loss. Lisdexamfetamine increases synaptic dopamine and norepinephrine levels, influencing reward pathways and satiety signals. Topiramate modulates GABA and glutamate neurotransmission and upregulates anorexigenic pathways, including POMC/melanocortin signaling. Phentermine, a sympathomimetic amine, enhances norepinephrine activity in the hypothalamus to reduce appetite. GLP-1 receptor agonists delay gastric emptying and promote central satiety signaling. Setmelanotide, a melanocortin-4 receptor agonist, directly stimulates the MC4R pathway involved in energy homeostasis and appetite control. Metformin indirectly contributes to appetite reduction by improving insulin sensitivity and inhibiting hepatic glucose output.

The mechanisms underlying its weight-reducing effects are not fully understood but may involve enhanced hypothalamic expression of anorexigenic neuropeptides, including proopiomelanocortin POMC, corticotropin-releasing hormone CRH, and thyrotropin-releasing hormone TRH, possibly modulating the POMC/melanocortin-4 receptor (MC4R) pathway [21]. Additional proposed mechanisms include inhibition of voltage-gated sodium channels, glutamate receptors (AMPA/kainate), carbonic anhydrase, and potentiation of GABAergic activity—all of which may contribute to reduced appetite and food intake (Figure 1) [22,23,24].

Clinical studies have shown that topiramate can be effective in managing obesity in children. In a case series of five children under 12 years of age (mean age: 10.3 years), use of topiramate resulted in a 12% reduction in the BMI% of the 95th percentile (also referred to as the extended BMI percentile) after 16 weeks [22]. Caregivers also reported decreased appetite and less impulsive eating behavior [22]. These findings suggest potential benefits for younger patients; however, the available data are currently limited.

Topiramate is usually initiated at a dose of 25 mg daily, taken in the morning, and increased by 25 mg each week as needed. Effective doses for weight management are usually between 50 and 100 mg/day. If the response is inadequate, the dose may be increased. The medication can be given in the evening if patient experiences drowsiness, fatigue, or cognitive decline. Discontinuation should be considered if appetite suppression or weight stabilization does not occur after 3–4 months at higher doses.

Lower doses are well tolerated, though the incidence of side effects rises with an increase in the dose. Reported adverse effects include paresthesia, reduced sweating that may lead to overheating, cognitive slowing, somnolence, nephrolithiasis, and metabolic acidosis [25]. Rare adverse effects are acute-angle closure glaucoma and serious skin reactions such as Stevens–Johnson syndrome [26]. Topiramate is associated with significant teratogenic risks, including increased rates of oral clefts and neurodevelopmental disorders, when used during pregnancy, especially in the first trimester [27]. Post-pubertal females taking topiramate should be counseled about these risks and advised to use effective contraception to prevent pregnancy during treatment. Topiramate should be tapered gradually to avoid seizure risk, even in patients without epilepsy [28].

### 2.2. Lisdexamfetamine

Lisdexamfetamine is a long-acting stimulant approved for the treatment of attention-deficit/hyperactivity disorder (ADHD) in children over six and binge eating disorder in adults [29,30,31]. As a prodrug, it requires enzymatic conversion in the bloodstream to its active form, d-amphetamine. This pharmacokinetic profile results in a delayed onset of action and lower peak plasma concentrations, contributing to a lower potential for abuse [32]. Its active metabolite, dextroamphetamine, increases synaptic levels of dopamine and norepinephrine. These neurotransmitters modulate appetite, satiety, and reward pathways, ultimately leading to appetite suppression (Figure 1) [31]. A study showed lisdexamfetamine significantly lowered BMI z scores in children with ADHD and overweight status or obesity. The mean BMI z score decreased by −0.41 (95% CI: −0.57 to −0.25; *p* < 0.001) in children with severe obesity and by −0.44 (95% CI: −0.56 to −0.31; *p* < 0.001) in those with mild-to-moderate obesity [33]. Notably, younger children aged 4–10 years demonstrated greater reductions in BMI, particularly within the overweight and mild-to-moderate obesity categories [33]. In a case series of five children with severe obesity, the median age at treatment initiation was 6.5 years (range, 4.5–14 years). The BMI% of the 95th percentile significantly decreased by 24% after 12 months of treatment. Four of the five children were young children (age range, 4.5–10 years) [34].

Common side effects include decreased appetite, weight loss, and insomnia [35,36,37,38,39]. In a two-year open-label trial involving 191 children with ADHD, decreased appetite (49.4%), weight loss (18.2%), insomnia (13.1%), and irritability (8.6%) were most frequent, mainly occurring early in treatment [39]. Preschool-aged children showed similar tolerability in a 52-week study; decreased appetite was the only adverse effect in over 10% of participants, and no serious events were reported [36]. Changes in blood pressure and pulse were minimal and not clinically significant [36].

The initial dose is 20–30 mg daily, increasing by 10 mg every 2–3 months as needed. The maximum recommended dose is 70 mg per day. If there is no evidence of appetite suppression or if weight gain persists despite titration after a period of 12 weeks, the medication should be discontinued (Table 1).

### 2.3. Glucagon-like Peptide 1 (GLP-1) Receptor Agonists

Glucagon-like peptide-1 (GLP-1) receptor agonists mimic the effects of endogenous GLP-1, a hormone secreted by intestinal L-cells in response to nutrient intake. GLP-1 binds to receptors in tissues like the hypothalamic appetite-regulation centers of the brain and pancreatic β-cells, enhancing insulin secretion in a glucose-dependent manner (Figure 1) [61]. GLP-1 receptor agonists also delay gastric emptying, thereby promoting satiety; activate the hindbrain to centrally modulate appetite; and influence reward pathways to reduce pleasure-driven eating (Figure 1) [62]. Additionally, they provide cardioprotective and renal protective benefits in adults [63,64]. GLP-1 receptor agonists are not recommended in patients with a family history of medullary thyroid carcinoma or multiple endocrine neoplasia type 2A or 2B, due to an association between GLP-1 receptor agonists and benign and malignant thyroid C cell tumors in rodents [49]. Additionally, stimulation of calcitonin release has been reported in rodents exposed to liraglutide and exenatide [65].

In a phase 3a, double-blind, randomized, placebo-controlled trial involving 82 children with non-syndromic obesity (SCALE Kids, ages 6 to <12) liraglutide (initial dose of 0.6 mg daily and increased weekly over a maximum of 8–10 weeks to 3 mg or the maximum tolerated dose; n = 56) led to a mean BMI reduction of –5.8% at week 56, while those receiving placebo (n = 26) saw a 1.6% increase—a difference of –7.4 percentage points (95% CI: –11.6 to –3.2; *p* < 0.001) [51]. Gastrointestinal adverse effects occurred more often with liraglutide (80% vs. 54%), although overall adverse event rates were similar (89% vs. 88%) [51]. Another short-term, randomized, double-blind, placebo-controlled trial in children (mean age 9.9 ± 1.1 years, n = 24) found that 56.3% of liraglutide recipients reported mainly gastrointestinal symptoms and asymptomatic hypoglycemia [66]. Currently, GLP-1 receptor agonists are not approved for the treatment of obesity in children under 12 years of age. The use of GLP-1 agonists in children under 12 years of age should be considered on a case-by-case basis due to limited data and the absence of FDA approval.

### 2.4. Metformin

Metformin is the recommended first-line therapy for metabolically stable children aged 10 and above with type 2 diabetes mellitus [67]. In children and adolescents with obesity, metformin may lead to modest weight reduction in some cases [68,69]. In a randomized, double-blind, placebo-controlled trial involving 100 children with severe obesity (6–12 years), alongside lifestyle interventions conducted for 6 months, the metformin group showed a greater reduction in BMI (difference between metformin and placebo groups −1.09 ± 0.38 kg/m^2^, *p* = 0.006) and BMI z-score (difference between metformin and placebo groups −0.07 ± 0.03, *p* = 0.02) [42]. Improvements were also noted in fasting insulin and insulin resistance (HOMA-IR), although no significant differences in glucose levels were observed [42]. The medication was well tolerated, with mild gastrointestinal side effects being the most commonly reported [42].

In a systematic review of 24 randomized controlled trials (age 4–19 years), metformin resulted in modest decreases in BMI (range of mean values: −2.70 to 1.30 vs. −1.12 to 1.90), BMI *z* score (range of mean values: −0.37 to −0.03 vs. −0.22 to 0.15), and homeostatic assessment of insulin resistance (range of mean values: −3.74 to 1.00 vs. −1.40 to 2.66) [69]. The mechanism of action for weight reduction with metformin is not fully understood. Metformin reduces hepatic glucose production, decreases intestinal glucose absorption, and increases peripheral insulin sensitivity, and may reduce appetite by raising glucagon like peptide-1 levels. Although it is not approved by the FDA for weight management in the pediatric population, metformin is commonly used off-label for this indication in clinical practice.

Metformin should be started at a dose of 500 mg once daily. The dose may be increased by 500 mg per day on a weekly basis, up to a maximum of 2000 mg daily (administered as 1000 mg twice daily), or less, as limited by the highest tolerated and effective dose, especially in younger children (Table 1) [42]. Extended-release formulations may be considered to improve tolerability and compliance in those who have difficulty adhering to a twice-daily immediate-release regimen. Metformin should be taken with food, to minimize gastrointestinal side effects.

Metformin can decrease the absorption of vitamin B12 and folate; therefore, patients should be advised to take a daily multivitamin supplement [43]. Metformin is contraindicated in those with cardiopulmonary insufficiency, mitochondrial disorders, cirrhosis, impaired renal function or hepatitis, due to the elevated risk of lactic acidosis [43]. In the context of elective surgical procedures, metformin should be discontinued 24 h prior to the intervention and resumed 48 h postoperatively, provided that renal function remains stable [70].

### 2.5. Setmelanotide

Setmelanotide is a melanocortin-4 receptor (MC4R) agonist approved to treat genetic obesity due to pro-opiomelanocortin (POMC), proprotein convertase subtilisin/kexin type 1 (PCSK1), or leptin receptor (LEPR) deficiencies, as well as Bardet–Biedl syndrome (BBS), in patients aged 2 years and older [45,46,47].

The leptin–melanocortin signaling pathway plays a central role in regulating hunger and satiety [71]. Setmelanotide works by binding to MC4R receptors and subsequently increasing satiety and energy expenditure [72]. Pathogenic homozygous or compound heterozygous variants in genes encoding components of this pathway—including MC4R, POMC, LEP (leptin), and LEPR—can result in early-onset severe obesity [72]. Bardet–Biedl syndrome, a ciliopathy with autosomal recessive inheritance, is frequently associated with hyperphagia and obesity, in addition to syndromic features such as polydactyly, retinal dystrophy, and renal abnormalities [73].

Phase III trials of setmelanotide showed significant weight loss and hunger reduction in individuals with POMC and LEPR deficiency. After 12 months, 80% (8/10) of the POMC group and 45% of the LEPR group (5/11) achieved at least a 10% weight loss, with a mean hunger-score decrease of 27.1% in the POMC group and 43.7% in the LEPR group [16]. Patients with Bardet–Biedl syndrome had a mean body-weight change of −11.3% at 6 months (90% CI: −15.5% to −7.0%; n = 8), and −16.3% at 12 months (90% CI: −19.9% to −12.8%; n = 7), alongside continued reductions in hunger scores [17]. In an open-label, phase 3, multicenter study involving 11 children aged 2–5 years with biallelic POMC, PCSK1, or LEPR variants, or genetically confirmed Bardet–Biedl syndrome (BBS), 83% achieved a ≥0.2-point reduction in BMI Z score over 52 weeks, with a mean BMI reduction of 18% observed overall (26% in the POMC/LEPR group and 10% in the BBS group) [45]. Ninety-one percent of caregivers reported reduced hunger in patients, and mild-to-moderate adverse events, including hyperpigmentation and gastrointestinal symptoms, were reported [45].

Setmelanotide is administered once daily by subcutaneous injection. For children aged 2 to 6 years, the typical starting dose is to 0.5 mg per day for 2 weeks. The recommended initial dose for those 6 to 12 years old is 1 mg daily, and 2 mg daily is the recommended initial dose for those who are 12 years of age and older. The dose may be adjusted up to a maximum of 3 mg per day, based on clinical response and tolerability (Table 1) [16,45]. Common side effects are injection site reactions, nausea, abdominal discomfort, flu-like symptoms, and skin hyperpigmentation [48].

## 3. Pharmacological Treatment of Pediatric Obesity in Children Aged ≥ 12 Years

Several pharmacological agents are approved for weight management in children and adolescents aged 12 years and older. Recently approved medications include liraglutide, a glucagon-like peptide-1 (GLP-1) receptor agonist (FDA approved 2020); semaglutide (approved 2022); and the extended-release combination phentermine and topiramate (Qsymia, approved 2022) [15]. The available studies are limited to short-term results, and there are little data on the long-term safety or sustained weight loss after discontinuation of the medication, leading to concerns about possible weight regain [74]. Patients and families should be thoroughly counseled about the potential side effects, lack of long-term safety data, and possibility of weight regain. These medications should be prescribed only as an adjunct to intensive lifestyle interventions aiming to optimize outcomes [7].

### 3.1. GLP-1 Receptor Agonists

Liraglutide and semaglutide are approved for weight management in children 12 years and older, with semaglutide showing greater effectiveness. The once-weekly dosing for semaglutide improves adherence.

#### 3.1.1. Semaglutide

Semaglutide was approved by the FDA in December 2022 for obesity in adolescents aged 12 and older. [75]. The starting dose for weight management is 0.25 mg weekly, increasing as tolerated up to 2.4 mg (0.5 mg weekly × 4 weeks, then 1 mg weekly × 4 weeks, then 1.7 mg weekly × 4 weeks, then 2.4 mg weekly × 4 weeks). If side effects occur at higher doses, the medication dose should be lowered to the previously tolerated dose (Table 1).

In a placebo-controlled trial, weekly subcutaneous semaglutide led to larger reductions in BMI (−16.1% with semaglutide and +0.6% with placebo), body weight (−15.3 kg with semaglutide and +2.4 kg with placebo), waist circumference, glycated hemoglobin levels, lipid parameters (excluding HDL), and alanine aminotransferase at 68 weeks versus placebo [14]. Semaglutide recipients also reported improved quality of life, mainly due to better physical comfort [14].

There are concerns that GLP-1 receptor agonists may perform differently in real-world settings, compared to clinical trials. In an observational study from the United Kingdom of 50 children treated with semaglutide for at least six months, the mean total weight loss was 6.4 ± 6.3% (*p* < 0.001) [76] at 6 months. BMI SDS decreased by −0.32 ± 0.27 (*p* < 0.001) and body weight dropped by −7.03 ± 7.50 kg at 6 months (*p* < 0.001) [76]. At 12 months, 14 patients showed a mean weight loss of 8.9 ± 10.0% (*p* < 0.001) [76]. Notably, nine patients experienced no weight loss, thereby highlighting the heterogeneity in weight loss associated with these medications [76].

#### 3.1.2. Liraglutide

Liraglutide is approved by the U.S. FDA for treating obesity in adolescents aged 12 years and older, and for the management of type 2 diabetes in children aged 10 years and older [52].

In a randomized controlled trial with adolescents aged 12 to <18 years with obesity, liraglutide 3 mg over 56 weeks resulted in a mean treatment difference in BMI of −4.6% (−4.2 ± 0.66 with liraglutide and 0.35 ± 0.99) and in BMI standard deviation score (SDS) of −0.22. There were no significant changes observed in cardiometabolic risk markers or quality of life measures [50]. After treatment discontinuation, a greater increase in the BMI SDS was observed with liraglutide than with placebo (0.22 vs. 0.07; estimated treatment difference, 0.15; 95% CI, 0.07 to 0.23) [50].

The initial dose is 0.6 mg once daily. The dose may be increased by increments of 0.6 mg each week to reach a maximum of 3 mg once daily. If side effects occur at a higher dose, the medication dose may be reduced to the previously tolerated dose. Liraglutide use has declined due to the requirement for daily injections and its lower weight-loss effect compared to semaglutide.

The most commonly reported adverse effects of GLP-1 receptor agonists are nausea, vomiting, abdominal pain, diarrhea, and headache [14,50]. In a double-blind, randomized, placebo-controlled clinical trial, 5% of adolescents in the semaglutide group and 4% in the placebo group discontinued the treatment, due to mainly gastrointestinal adverse events. Cholelithiasis occurred in 4% of the semaglutide group, versus no incidence in the placebo group [14].

The strategies to mitigate the gastrointestinal side effects include consuming small, frequent meals and avoiding high-fat or greasy foods. For managing diarrhea, it is beneficial to limit or temporarily avoid isotonic and hypertonic beverages, fruit juices, high-fiber foods, and artificial sweeteners [77]. Constipation may be alleviated through increased dietary-fiber and fluid intake, and regular physical activity. Should symptoms persist despite these measures, short-term symptomatic pharmacotherapy targeting the specific gastrointestinal disturbance may be warranted [78]. Nutritional priorities for patients taking glucagon-like peptide-1 (GLP-1) receptor agonists should also center on maintaining nutrient adequacy and muscle mass and supporting long-term metabolic health. Appetite suppression and early satiety induced by GLP-1 receptor agonists can lead to reduced caloric and protein intake, increasing the risk of micronutrient deficiencies (notably vitamin D, B12, calcium, iron, magnesium, and zinc) and sarcopenia, especially during rapid or substantial weight loss. Periodic laboratory assessment and consideration of multivitamin supplementation are recommended for those with significant appetite reduction or weight loss [79,80]. Structured resistance training and prioritization of protein-rich foods (e.g., fish, eggs, dairy, legumes, and nuts/seeds) are essential to preserve muscle and bone mass [79,80]. Close monitoring and regular follow-up with a dietitian are essential, particularly for adolescents at risk of eating disorders, disordered eating behaviors, or body image disturbances [77].

A key issue with GLP-1 receptor agonists is weight regain and reversal of improvements in cardiometabolic risk factors after discontinuation of these medications [50,81,82]. Consistent data on long-term use in children are limited, though adult studies report discontinuation rates between 37% and 81% at one year due to factors like cost, insurance, comorbidities, and absence of type 2 diabetes [83,84,85]. All individuals prescribed GLP-1 receptor agonists should also receive intensive, multicomponent behavioral interventions—including a nutrient-dense, reduced-calorie diet; structured physical activity; and personalized strategies to support weight loss and maintenance [79]. Continuous lifestyle support is recommended to reduce weight regain after discontinuation of GLP-1 receptor agonist therapy. Further research is needed on the impact of GLP-1 receptor agonists on disordered eating and eating disorders [86,87].

### 3.2. Phentermine

Phentermine, a sympathomimetic amine that suppresses appetite, was approved by the FDA in 1959 for short-term use (≤12 weeks) in adolescents over 16 years [75,88]. Phentermine is usually prescribed at a dose ranging from 15 mg to 37.5 mg per day. Combined with lifestyle therapy in adolescents, phentermine led to significantly greater body-weight and BMI reductions than lifestyle therapy alone at 1 month (−1.4 kg, −1.6%), 3 months (−2.6 kg, −2.9%), and 6 months (−3.2 kg, −4.1%) [56]. Reported side effects include insomnia, irritability, anxiety, and slight increases in blood pressure and heart rate [55]. However, studies there is no evidence to suggest a higher risk of major cardiovascular events [89].

### 3.3. Phentermine/Topiramate ER

In a randomized, double blind trial involving adolescents aged 12 to <17 years with obesity, mid- and high-dose phentermine/topiramate extended-release (ER) (7.5 mg/46 mg and 15 mg/92 mg) led to significant BMI reductions of −8.11% and −10.44%, respectively [15]. Both groups also demonstrated significant improvements in triglyceride levels and high-density lipoprotein cholesterol (HDL-C) after 56 weeks [15]. Adverse events in the high-dose group included biliary stones, depression, and suicidal ideation, with psychiatric symptoms persisting up to 105 days post-treatment [15]. Counseling female adolescents on pregnancy prevention is critical due to teratogenicity risks [75]. Phentermine/topiramate extended release is available in four dosage strengths: 3.75/23 mg, 7.5/46 mg, 11.25/69 mg, and 15/92 mg. The initial dose of phentermine–topiramate is 3.75/23 mg for 14 days, followed by 7.5/46 mg thereafter. The dose can be increased to 11.25/69 mg for 14 days and then to 15/92 mg daily, if after 12 weeks of the 7.5/46 mg, improvements in body weight and BMI are not achieved (Table 1). The medication should be discontinued if there is no improvement in BMI or weight after 12 weeks on the highest dose.

### 3.4. Topiramate

Though topiramate alone is not approved by the FDA as monotherapy for obesity in children, it was one of the commonly prescribed medications before the GLP-1 receptor agonists became available [90]. Studies in adolescents have demonstrated that topiramate, when combined with lifestyle interventions, results in a modest reduction in body weight [41,91]. A large retrospective study involving 282 youths showed that topiramate combined with lifestyle interventions led to a 3.4% reduction in BMI, and a reduction in BMI% of the 95th percentile by 9.3%, after 12 months [41]. A randomized trial with adolescents aged 12–18 years with severe obesity evaluated the efficacy of topiramate in maintaining weight loss following a 4-week meal-replacement therapy [91].There was no statistically significant difference in percent BMI change between the topiramate and placebo groups after 28 weeks. However, the topiramate group demonstrated a reduction in visceral adiposity and lower very-low-density lipoprotein cholesterol (VLDL-C) levels, with no adverse effects [91].

Counseling pregnancy prevention is essential due to the teratogenic effects of topiramate. At high doses, topiramate may also decrease the effectiveness of oral combined hormonal contraceptives and therefore, alternative or additional contraceptive methods should be offered. The medication should be gradually tapered off upon discontinuation to avoid the occurrence of seizures.

### 3.5. Orlistat

Orlistat was approved by the FDA for weight loss in 12–18-year-olds in 2003. Its use is commonly associated with oily stools, flatulence, fecal urgency, and incontinence. Orlistat is a gastrointestinal lipase inhibitor, and it reduces the absorption of dietary fat by about one-third. The recommended dose is 120 mg three times daily with meals [59]. In a randomized controlled trial, treatment with orlistat 120 mg three times daily resulted in a mean BMI reduction of 0.55 kg/m^2^, whereas the placebo group experienced a mean increase of 0.31 kg/m^2^ [58]. Additionally, 26.5% of participants receiving orlistat achieved at least a ≥5% reduction in BMI, compared to 15.7% in the placebo group. The adverse effects reported were symptoms primarily gastrointestinal in nature, including fecal urgency, fecal incontinence, and oily or fatty stools. Its modest efficacy and the prevalence of adverse effects limit the long-term use of orlistat as a therapeutic option in adolescents.

### 3.6. Metformin

Metformin is the first-line treatment for type 2 diabetes in adolescents and is frequently used off-label for obesity [90]. In a meta-analysis of 38 randomized controlled trials (2199 children and adolescents, mean age 13.7 years; dose 500–3000 mg; duration 12–192 weeks), metformin was associated with a mean reduction in BMI of 1.1 units versus controls [92]. Another study revealed that metformin-associated weight loss was most notable in individuals with Hispanic ethnicity, those with acanthosis nigricans, and those with a BMI under 35 kg/m^2^ [44].

## 4. The Future of Anti-Obesity Pharmacotherapy

Several clinical trials for new anti-obesity medications in adults are underway. These medications may be considered for future pediatric studies if the results in adults are promising. However, conducting clinical trials on anti-obesity medications in pediatric populations presents several unique challenges. Ethical considerations are paramount, as children are a vulnerable group, requiring careful assessment of the risk–benefit ratios before exposing them to pharmacologic agents intended for weight management. Recruitment and retention can be difficult due to parental hesitancy. Physiological differences across developmental stages make the interpretation of metabolic outcomes challenging. Additionally, long-term follow-up is often necessary to evaluate sustained efficacy and potential effects on growth and puberty, which can be logistically demanding. Given these challenges, most existing pediatric data are derived from retrospective studies rather than controlled prospective trials. Interpreting findings from such designs can be difficult, as they are more susceptible to bias and confounding, and feature small sample sizes, limiting the ability to establish causality. However, these studies remain valuable for generating hypotheses and informing real-world clinical practice until more robust randomized controlled data become available. In this section, we aim to summarize medications that have demonstrated efficacy in adults and may be considered for future clinical studies in pediatric populations to evaluate their safety and effectiveness in younger age groups.

Trials investigating newer potential anti-obesity medications, including co-agonists and tri-agonists that target peptides such as GLP-1, glucose-dependent insulinotropic polypeptide (GIP), amylin, and glucagon are underway. Tirzepatide, a dual GIP/GLP-1 receptor agonist, is approved for obesity and type 2 diabetes in adults [93,94]. Randomized placebo-controlled trials have shown that tirzepatide leads to significant weight loss. In the highest dosage group (15 mg), 88.1%, 63.3%, and 51.8% of patients lost at least 5%, 10%, and 15% of body weight, respectively. In addition, tirzepatide improves cardiometabolic risk factors, including hemoglobin A1c, waist circumference, body mass index, and lipid profiles, and demonstrates a favorable safety profile [94].

Dual GLP-1/glucagon receptor agonists (GLP-1/Gcg RAs) promote weight loss by activating both the GLP-1 and glucagon receptors. This dual mechanism enhances weight reduction while minimizing the risk of hyperglycemia seen with glucagon receptor activation alone [95]. In a phase 2 trial, survodutide, an injectable dual glucagon/GLP-1 receptor agonist, produced significant mean weight losses: −6.2%, −12.5%, −13.2%, and −14.9% at 0.6 mg, 2.4 mg, 3.6 mg, and 4.8 mg, respectively, compared to −2.8% in the placebo group [96].

In a phase 2b randomized trial involving adults with type 2 diabetes, cotadutide—another glucagon/GLP-1 receptor agonist—demonstrated significant improvements in glycemic control and body weight, compared to placebo (*p* < 0.001 for all measures). The study also reported favorable changes in lipid parameters, alanine aminotransferase (ALT), aspartate aminotransferase (AST), procollagen type III peptide, and metabolic dysfunction-associated steatotic liver disease (MASLD) scores [97]. A phase 2 trial involving 338 adults with overweight status or obesity evaluated retatrutide—a triple agonist for GLP-1, GIP, and glucagon receptors—for weight loss over 48 weeks. Compared to a −2.1% reduction in the placebo group, mean weight losses with retatrutide were −8.7%, −17.1%, −22.8%, and −24.2% at doses of 1 mg, 4 mg, 8 mg, and 12 mg, respectively [98].

Oral semaglutide is not yet FDA-approved for obesity, but the data indicate that oral semaglutide 50 mg daily is associated with clinically significant weight loss. In a randomized double-blind, placebo-controlled, phase 3 trial of adults with overweight or obesity without type 2 diabetes, estimated mean body weight change from baseline to weak 68 was −15.1% (SE 0.5) in the oral semaglutide group and −2.4% (0.5) in the placebo group [99]. Phase 2 and phase 3 trials have also indicated that orforglipron, an oral, non-peptide GLP-1 receptor agonist, is associated with weight loss and improvement in cardiometabolic parameters in adults with obesity or in those with overweight status and at least one weight-related comorbidity, in the absence of diabetes [100,101].

## 5. Conclusions

Pharmacotherapy for pediatric obesity has advanced rapidly in recent years, offering new hope for children and adolescents who do not achieve sufficient weight reduction with lifestyle interventions alone. Preventing childhood obesity is critical not only for reducing immediate health risks but also for mitigating long-term consequences such as type 2 diabetes, cardiovascular disease, and psychosocial challenges that often persist into adulthood. Though lifestyle interventions remain the mainstay of the management of obesity in children, these medications provide additional options for selected patients requiring further support. Despite these advances, conducting clinical trials in pediatric populations is challenging, and important research gaps remain. Long-term safety and efficacy data are limited, especially for children under 12 years and for diverse populations. There is a need for studies evaluating outcomes beyond BMI reduction, such as body composition, psychosocial well-being, and quality of life. Additionally, optimizing access and equity in pharmacotherapy, as well as integrating these treatments into comprehensive care models, are critical areas for future investigation. The ongoing development of new medications, including incretin co-agonists and tri-agonists, holds promise for treatments of even greater efficacy and individualized therapies in the coming years.

## Figures and Tables

**Table 1:** Pharmacological treatment options for pediatric obesity.

Drug Name	Age	Weight Outcome	FDA-Approved Indication	Dose Titration	Comment	Potential Side Effects
Topiramate	<12 years	In a case series of 5 children, 12% reduction in BMI% of the 95th percentile after 16 weeks [22].	Not FDA-approved for obesity as monotherapy	Titrate weekly; typical doses are 25–100 mg daily or maximum tolerated effective dose [40].	Should be tapered gradually to avoid seizure risk, even in patients without epilepsy [28].	Paresthesia, cognitive slowing, somnolence, nephrolithiasis and metabolic acidosis [25]. The rare adverse effects are acute-angle closure glaucoma and serious skin reactions such as Stevens–Johnson syndrome [26].
	Adolescents	A 3.4% reduction in BMI and decrease in the BMI% of the percentile by 9.3% after 12 months of therapy [41].				Teratogenic risks, including increased rates of oral clefts and neurodevelopmental disorders.
Lisdexamfetamine	<12 years	Case series of five children with severe obesity, (median age 6.5 years). Decrease in the BMI% of the 95th percentile by 24% after 12 months [34]. In children with ADHD, BMI z score decreased by –0.41 in severe obesity group and by −0.44 in mild-to-moderate obesity group [33].	FDA-approved for ADHD in children ≥ 6 y; weight management is off-label	Start 20–30 mg daily; titrate based on response (max 70 mg daily) [40].	Contraindicated in children with history of substance abuse, symptomatic cardiovascular disease, hypertension, hyperthyroidism, or with MAOI use within 14 days. Must be used cautiously in those with structural heart disease or psychiatric disorders.	Decreased appetite, insomnia, irritability, increased heart rate, and anxiety [39].
Metformin	<12 years	RCT: Children (6–12 years) with severe obesity plus lifestyle interventions, decreased BMI z-score (−0.07 ± 0.03, *p* = 0.02) compared to placebo after 6 months [42].	Children ≥ 10 y with T2DM	Start 500 mg daily; titrate to 1500–2000 mg daily or maximum tolerated effective dose [42].	Contraindicated in cases of severe renal impairment (eGFR < 30 mL/min/1.73 m^2^), cardiopulmonary insufficiency, mitochondrial disorders, cirrhosis, hepatitis, or a history of alcohol use disorder, in addition to cases of hypersensitivity to metformin [43].	GI disturbance, vitamin B12 deficiency, and lactic acidosis [43].
	Adolescents	Systematic review: decrease in BMI by 1.16 kg/m^2^ after 6 mo [44].				
Setmelanotide		POMC/PCSK1 deficiency: weight change −25.6% at 1 y; LEPR deficiency: 45% lost >10% body weight at 1 y; Bardet–Biedl syndrome: weight change −16.3% at 12 months [16,17,45].	≥2 y with pathogenic/likely pathogenic/uncertain variants in LEPR, POMC, or PCSK1 deficiency; Bardet–Biedl syndrome [45,46,47]	Ages 2–6: start 0.5 mg daily; 6–12: start 1 mg daily; >12: start 2 mg daily. Titrate up to 3 mg daily or down to 0.5 mg based on response and tolerability [16,45].		Injection site reactions, skin hyperpigmentation, rash, alopecia, GI disturbance, and flu-like symptoms [48].
Semaglutide	Adolescents	RCT: mean BMI reduction by 16.1% from baseline vs. 0.6% in placebo [14].	Weight management in adolescents ≥12 y; weekly dosing 0.25–2.4 mg	Start 0.25 mg weekly; titrate every 4 weeks to max 2.4 mg weekly or maximum tolerated effective dose [14].	Contraindicated in patients with a personal or family history of MEN 2A or 2B or medullary thyroid carcinoma [49].	Gastrointestinal disturbance (nausea, diarrhea, and vomiting), headaches, hypoglycemia, cholelithiasis, and pancreatitis [14,50].
Liraglutide	<12 years	RCT: a mean BMI reduction of –5.8% at week 56 [51].	Not approved			
	Adolescents	RCT: mean BMI difference of −4.6% (−4.2 ± 0.88 with liraglutide and 0.35 ± 0.91 in placebo) [50].	T2DM in adolescents ≥ 10 y (0.6–1.8 mg daily); weight management in adolescents ≥ 12 y (0.6–3 mg daily) [52]	Start 0.6 mg daily; increase weekly to target dose (max 3 mg) or maximum tolerated effective dose [50].		
Phentermine/Topiramate	Adolescents	RCT: mid dose BMI change −8.11%; high dose −10.44% [15].	Weight management in adolescents ≥ 12 y	Start with 3.75/23 mg daily for 14 days, then 7.5/46 mg daily. If inadequate weight/BMI improvement after 12 weeks, titrate to 11.25/69 mg for 14 days, then to 15/92 mg daily [15].	Contraindicated in hyperthyroidism, pregnancy, glaucoma, cardiac disease; avoid within 2 wk. of MAOI use [28,53,54].	Topiramate: previously discussed. Phentermine: Dry mouth, insomnia, irritability, slightly increased heart rate, increased blood pressure, constipation, and anxiety [55].
Phentermine	Adolescents	BMI decreased by 4.1% at 6 months [56].	Short-term weight management in adolescents ≥ 17 y (short-term)	Start 15 mg daily; adjust based on response and tolerability, dose ranging from 15 mg to 37.5 mg per day [40].	Contraindicated in hyperthyroidism, uncontrolled hypertension, cardiac disease, history of drug abuse and pregnancy [57].	Previously discussed.
Orlistat		RCT: BMI change −0.55 kg/m^2^ vs. +0.31 in placebo group [58].	Weight management in adolescents ≥ 12 y	120 mg three times daily with meals containing fat [59].	Contraindicated in cholestasis.	Abdominal discomfort, flatulence, and fat-soluble vitamin deficiency [60].

Abbreviations: ADHD, attention-deficit/hyperactivity disorder; BMI, body mass index; eGFR, estimated glomerular filtration rate; GI, gastrointestinal; LEPR, leptin receptor; MAOI, monoamine oxidase inhibitor; PCSK1, proprotein convertase subtilisin/kexin type 1; POMC, pro-opiomelanocortin; RCT, randomized controlled trial; T2DM, type 2 diabetes mellitus.

## Data Availability

No new data were created or analyzed in this study.
